# Effect of Post-stroke Depression on Functional Outcomes of Patients With Stroke in the Rehabilitation Ward: A Retrospective Cohort Study

**DOI:** 10.1016/j.arrct.2023.100287

**Published:** 2023-08-02

**Authors:** Yoshitaka Wada, Yohei Otaka, Taiki Yoshida, Kanako Takekoshi, Raku Takenaka, Yuki Senju, Hirofumi Maeda, Seiko Shibata, Taro Kishi, Satoshi Hirano

**Affiliations:** a Department of Rehabilitation Medicine Ⅰ, School of Medicine, Fujita Health University, Toyoake, Japan; b Faculty of Rehabilitation, School of Health Sciences, Fujita Health University, Toyoake, Japan; c Department of Nursing, Fujita Health University Hospital, Toyoake, Japan; d Department of Psychiatry, School of Medicine, Fujita Health University, Toyoake, Japan

**Keywords:** Activities of daily living, Cerebrovascular disorders, Comorbidity, Depression, Rehabilitation

## Abstract

**Objective:**

To investigate the prevalence of post-stroke depression in a rehabilitation ward and elucidate its effect on functional improvement and outcomes.

**Design:**

Retrospective cohort study.

**Setting:**

A convalescent rehabilitation ward at a University Hospital.

**Participants:**

A total of 114 patients with stroke (mean [SD] age, 67.2 [13.5] years; men, 76) assessed at 2 weeks after admission using the Mini-International Neuropsychiatric Interview were enrolled.

**Main Outcome Measure:**

Functional independence measure (FIM) efficiency during hospitalization in the ward.

**Results:**

Eleven patients (9.6%) had depression based on the Mini-International Neuropsychiatric Interview. Total FIM efficiency and FIM efficiency in the subtotal of motor items were significantly higher in the non-depression group than in the depression group (median [interquartile range]: 0.69 [0.39-0.95] vs 0.41 [0.24-0.63], *P*=.027; and 0.56 [0.38-0.80] vs 0.42 [0.18-0.49], *P*=.023, respectively). Patients in the non-depression group had higher FIM scores at discharge (median [interquartile range]: 116.0 [104.5-123.0] vs 104.0 [82.5-112.0], *P*=.013, respectively), and were more likely to be discharged home (80.6% vs 36.4%, *P*=.003). Furthermore, patients in the depression group also stayed significantly longer in the ward (71.0 [36.1] vs 106.1 [43.3], *P*=.010).

**Conclusions:**

Patients with post-stroke depression showed poorer efficiency of functional recovery than those without depression. A future multicenter study with a larger sample size is needed to verify these findings.

Stroke is a major cause of death and disability worldwide, and post-stroke depression is a common clinical problem affecting approximately 30% of patients after stroke.^1^^,^^2^ Depression after stroke is associated with a reduced quality of life,^3^ concurrent with increased mortality,^4^ and caregiver stress.^5^ Furthermore, depression is generally associated with poorer functional outcomes after stroke.^6^ Therefore, in rehabilitation settings where the function of patients with stroke changes dramatically, clinicians should pay close attention to signs of depression. However, in a situation where the prevalence of depression varies with the length of time since stroke onset and the clinical setting,^6^ the effect of depression on functional improvement in rehabilitation settings remains inconclusive.

Several studies have previously reported depression to be associated with poor functional outcomes,^7^, ^8^, ^9^, ^10^ while other studies have shown no effect of depression on functional improvements in rehabilitation settings.^11^, ^12^, ^13^ One retrospective cohort study noted that patients with stroke with depression had significantly lower functional independence measure (FIM) scores at admission and discharge, and FIM gain than those without.^10^ Similarly, 2 studies^8^^,^^9^ using multivariable analyses reported that depression was associated with poor functional outcomes. In contrast, a few studies have found no differences in the Barthel index on admission or at 6 weeks, 6 months, and 12 months after discharge,^11^ or any improvement in Barthel index values over those recorded on admission.^12^^,^^13^ Thus, the influence of depression on functional outcomes after stroke in rehabilitation settings remains inconclusive.

To address these knowledge gaps, this retrospective cohort study aimed to investigate the prevalence of depression in patients with stroke admitted to a rehabilitation ward and elucidate the effect of depression on functional improvement and outcomes.

## Methods

### Study design and setting

This retrospective cohort study was conducted in the convalescent rehabilitation ward at Fujita Health University Hospital, Aichi, Japan. This study was approved by the Ethics Committee of Fujita Health University, and was conducted in accordance with the STROBE guideline.^14^ The requirement for informed consent was waived due to the retrospective study design, and therefore all individuals who did not opt out were included.

The setting of this study was a convalescent rehabilitation ward specialized for rehabilitation, and all patients were covered by the national medical insurance established in April 2000 in Japan. Patients who experienced stroke were cleared to stay in the ward for up to 180 days and could undergo a maximum of 3 h of rehabilitation treatment per day, consisting of physical therapy, occupational therapy, and speech-language therapy, as indicated. The rehabilitation program was tailored to the specific needs of each patient and included range of motion training, muscle strengthening training, gait training, and training for activities of daily living. The transdisciplinary team provided patients and their families with comprehensive rehabilitation, including the Mini-International Neuropsychiatric Interview (MINI)^15^, ^16^, ^17^ for depression screening. For patients who presented with depressive symptoms, the physician in charge of the patient judged whether to prescribe medication and consult a clinical psychologist or psychiatrist in addition to a team approach, including counseling by the nurse in charge of the patient.

### Participants

We enrolled patients hospitalized for stroke in the rehabilitation ward and discharged between January 2021 and July 2022 who underwent evaluation using the MINI at 2 weeks after admission. We only included patients with cerebral infarction and cerebral hemorrhage and excluded those with subarachnoid hemorrhage. We further included those with a previous history of stroke.

### Outcomes

The primary outcome in the present study was FIM efficiency^18^ during hospitalization in the ward. The secondary outcomes included total FIM score, subtotal FIM motor items score, subtotal FIM cognitive items score, gain in FIM scores, FIM effectiveness,^18^ length of ward stay, total rehabilitation time, and discharge destination. These outcomes, as well as the patients’ baseline characteristics, including age, sex, time from onset, Charlson Comorbidity Index^19^ as a comorbidity assessment, and Stroke Impairment Assessment Set as a comprehensive evaluation of motor impairments,^20^ were retrospectively collected from the medical records.

The FIM is a scale for activities of daily living consisting of 13 motor items and 5 cognitive items.^21^^,^^22^ The score for the subtotal motor items of the FIM ranges from 13 to 91, while that for the subtotal cognitive items of the FIM score ranges from 5 to 35: higher scores indicate better activities of daily living. Although recently replaced by “quality indicators” in the US, the validity and reliability of this scale have long been confirmed.^23^ FIM efficiency was calculated as follows: (FIM score at discharge – FIM score on admission)/length of hospital stay.^18^ FIM effectiveness was calculated as follows: (FIM score at discharge – FIM score on admission)/(126 – FIM score on admission).^18^ The gain in FIM was calculated as the total FIM score at discharge minus the total FIM score at admission.^18^ The FIM score was assessed on admission as well as at discharge by the therapists well-trained in scoring with the FIM in charge of patients.

The MINI is a short diagnostic structured interview for the Diagnostic and Statistical Manual (DSM)-III-R Axis I psychiatric disorders.^15^, ^16^, ^17^ This tool is compatible with the diagnostic criteria established by the DSM of mental disorders as well as the international classification of diseases.^24^^,^^25^ We employed the MINI for screening because a previous systematic review showed that the MINI fulfilled the minimum criteria for sensitivity and specificity for the diagnosis of depression.^26^ Questions were phrased to allow only yes or no answers, which made it easier for patients of all abilities, including severe stroke patients, to provide answers. A major depressive episode was identified as having 5 or more yes answers, including at least 1 A1 and A2. In the present study, depression was defined based on this major depressive episode. We used the Japanese version of the MINI,^27^ the score of which was determined 2 weeks after admission by the nurse in charge of the patients.

The Charlson Comorbidity Index scores comorbidities and evaluates 19 conditions related to chronic diseases, and the validity of this method has been confirmed.^19^ In the present study, the Charlson Comorbidity Index score was determined by a physiatrist based on the findings at the time of admission.

The length of ward stay was defined as the number of days from admission to discharge from the rehabilitation ward. The time of discharge was determined when the rehabilitation team judged that the patient had reached a plateau in activities of daily living. The total rehabilitation time (hours) was the time of rehabilitation during hospitalization in the convalescent rehabilitation ward.

The discharge destination was categorized as home, another hospital, or long-term care facility.

### Statistical analysis

Baseline characteristics were compared between the non-depression and depression groups using the Mann-Whitney *U* test or chi-square test, depending on the variable classification. We used G*power 3.1.9.6.^a^ to calculate the post hoc statistical power,^28^^,^^29^ setting the effect size to 0.5, the α error to 0.05, and the power (1-β error) to 0.455. The primary outcome, FIM efficiency, was compared between the non-depression and depression groups using the Mann-Whitney *U* test. The total FIM score, subtotal FIM motor items score, subtotal FIM cognitive items score, gain in the FIM scores, FIM effectiveness, and length of hospital stay were all compared using the Mann–Whitney U test. We further employed the Mann-Whitney *U* test for the functional outcome, as we took the non-normal distribution of the data into account. Discharge destinations were compared using the chi-square test. The effect size (ES) was calculated for each comparison of functional outcomes. The ES of the Mann-Whitney *U* test was calculated by dividing the *z* score by the square root of the total number of participants: ES=Z/sqrt (N). The ES of the χ2 test was calculated by dividing the χ2 value by the number of scores and taking the square root. Any *P* values less than .05 were considered as statistically significant. R version 4.1.0^b^ was used for all statistical analyses.

## Results

**Fig 1:**
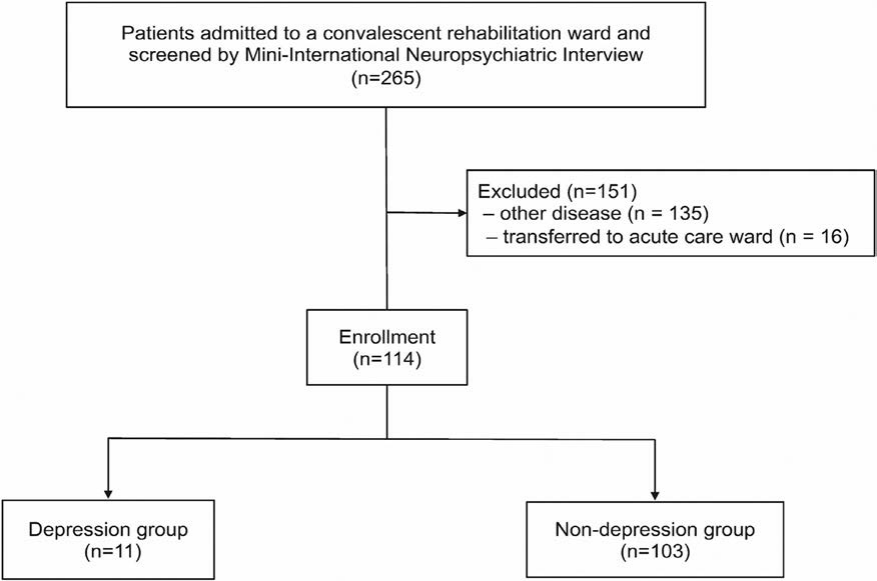
Flow diagram of patient selection.

A total of 114 stroke patients (76 men) were admitted to the ward during the study period, and screened with the MINI (fig 1). The participants’ characteristics are presented in table 1. The mean ± SD age of all participants was 67.2 (13.5) years. At admission to the rehabilitation ward, 11 patients (9.6%) had depression based on the MINI, 1 of whom had a medical history of depression. During hospitalization in the ward, 1 patient required consultation with a psychiatrist, and another patient was prescribed oral antidepressants by the physician-in-charge.

**Table 1:** Participants’ characteristics on admission to the rehabilitation ward

	Total (N=114)	Non-depression (n=103)	Depression (n=11)	*P* Value
Age, years, mean (SD)	67.2 (13.5)	67.5 (13.5)	63.9 (13.4)	.401
Sex, Men/Women, n	78/36	70/33	8/3	.999
Charlson Comorbidity Index, median (IQR)	1 (0-2)	1 (0-2)	0 (0-1.5)	.420
Previous history of depression, n	1	0	1	.097
Previous history of stroke, n	6	5	1	.464
Stroke type, cerebral infarction/cerebral hemorrhage, n	67/47	59/44	8/3	.521
Side of weakness, right/left/bilateral, n	58/49/7	55/43/5	3/6/2	.080
Time from onset, days, mean (SD)	23.3 (20.6)	22.3 (20.4)	30.1 (19.4)	.144
Stroke impairment assessment scale				
Subtotal motor items score, mean (SD)	12.7 (8.6)	12.7 (8.7)	10.5 (7.6)	.330
Knee-mouth, median (IQR)	3 (1-4)	3 (0.75-4)	3 (1-3)	.524
Finger function, median (IQR)	2 (0-4)	2.5 (0-4)	1 (1-3)	.409
Hip flexion, median (IQR)	3 (1-4)	3 (1-4)	2 (0.5-3.5)	.296
Knee extension, median (IQR)	3 (1-4)	3 (1-4)	3 (0-3.5)	.311
Foot pat, median (IQR)	3 (0-4)	3 (0-4)	2 (0-3.5)	.222

Abbreviations: IQR, interquartile range.

Regarding functional outcomes (table 2), we found no significant differences in FIM scores at admission (*P*>.05). However, at discharge, the total FIM score, subtotal FIM motor items score, and subtotal FIM cognitive items score were all significantly higher in the non-depression group than in the depression group (median [interquartile range]: 116.0 [104.5-123.0] vs 104.0 [82.5-112.0], *P*=.013, ES=0.230; 82.0 [74.0-89.0] vs 77.0 [53.5-80.5], *P*=.024, ES=0.210; and 34.0 [28.0-35.0] vs 29.0 (25.0-31.0), *P*=.027, ES=0.206, respectively). Furthermore, total FIM efficiency and FIM efficiency in the subtotal of motor items were significantly higher in the non-depression group than in the depression group (0.69 [0.39-0.95] vs 0.41 [0.24-0.63], *P*=.027, ES=0.206; and 0.56 [0.38-0.80] vs 0.42 [0.18-0.49], *P*=.023, ES=0.211, respectively). However, there was no significant difference in the FIM effectiveness or the FIM gain between groups (*P*>.05) (table 2).

**Table 2:** Functional outcomes of participants

	Total (N=114)	Non-depression (n=103)	Depression (n=11)	*P* Value	Effect Size
FIM on admission					
Subtotal FIM motor items score, median (IQR)	34.0 (17.0-52.8)	36.0 (18.0-54.5)	19.0 (15.0-36.5)	.066	0.171
Subtotal FIM cognitive items score, median (IQR)	24.0 (14.3-33.0)	24.0 (14.5-33.0)	19.0 (14.0-24.5)	.145	0.135
Total FIM score, median (IQR)	58.0 (39.5-82.5)	62.0 (41.5-86.0)	49.0 (29.0-59.0)	.073	0.166
FIM at discharge					
Subtotal FIM motor items score, median (IQR)	82 (73-89)	82.0 (74.0-89.0)	77.0 (53.5-80.5)	.024	0.210
Subtotal FIM cognitive items score, median (IQR)	34 (27.3-35.0)	34.0 (28.0-35.0)	29.0 (25.0-31.0)	.027	0.206
Total FIM score, median (IQR)	115.5 (101.3-121.8)	116.0 (104.5-123.0)	104.0 (82.5-112.0)	.013	0.230
FIM efficiency					
Subtotal of motor items, median (IQR)	0.53 (0.35-0.78)	0.56 (0.38-0.80)	0.42 (0.18-0.49)	.023	0.211
Total, median (IQR)	0.67 (0.37-0.88)	0.69 (0.39-0.95)	0.41 (0.24-0.63)	.027	0.206
FIM effectiveness					
Subtotal of motor items, median (IQR)	0.82 (0.65-0.94)	0.83 (0.66-0.95)	0.77 (0.34-0.83)	.058	0.176
Total, median (IQR)	0.81 (0.61-0.92)	0.82 (0.65-0.92)	0.67 (0.34-0.82)	.061	0.174
FIM gain					
Subtotal of motor items, median (IQR)	39.5 (20.3-51.8)	39.0 (20.5-51.0)	41.0 (21.5-56.5)	.777	0.026
Total, median (IQR)	42.0 (24.0-62.8)	42.0 (24.0-62.5)	45.0 (28.5-67.5)	.730	0.032
Length of ward stay, days, mean (SD)	74.3 (38.1)	71.0 (36.1)	106.1 (43.3)	.010	0.242
Total rehabilitation time, hour, mean (SD)	216.4 (114.9)	206.4 (109.5)	309.7 (121.6)	.010	0.242
Discharge destination					
Home, n	87	83	4	.003	0.283
Other hospitals or long-term care facilities, n	27	20	7		

Abbreviations: IQR, interquartile range.

Length of ward stay and total rehabilitation time were significantly shorter in the non-depression group than in the depression group (71.0 [36.1] vs 106.1 [43.3], *P*=.010, ES=0.242; 206.5 [109.5] vs 309.7 [121.6], *P*=.010, ES=0.242). In addition, the depression group showed a significantly lower rate of discharge to home compared with the non-depression group (36.4% vs 80.6%, *P*=.003, ES=0.283).

## Discussion

This study investigated the functional outcomes of patients with stroke with and without depression in the rehabilitation ward. Two weeks after admission to the rehabilitation ward, 9.6% of patients showed depression. Patients in the non-depression group showed greater FIM efficiency, indicating a decreased efficiency in improving activities of daily living in patients with depression. Additionally, patients in the depression group had significantly longer lengths of stay and total rehabilitation time in the rehabilitation ward and were further less likely to be discharged home.

The prevalence of depression after stroke in our study sample was lower than those of previous studies, which reported a prevalence range of 13%-57% in rehabilitation settings.^7^, ^8^, ^9^, ^10^, ^11^, ^12^, ^13^ We used the MINI to assess depression, while these previous studies^7^, ^8^, ^9^, ^10^, ^11^, ^12^, ^13^ used a variety of measures, including the Self-Rating Depression Scale, the Geriatric Depression Scale, the Hospital Anxiety and Depression Scale, and the Hamilton Depression Rating Scale. Thus, the inconsistency in the prevalence may be at least partially attributed to the differences in the tests used to identify depression. Although the prevalence of depression noted in the present study was relatively low compared with that seen in previous studies, the possibility of underestimation on depression diagnosis was low because the MINI is slightly more overinclusive in the diagnoses than the Structured Clinical Interview for DSM-III-R Patients or expert diagnosis.^27^ Second, this may indicate that the prevalence of PSD differs according to race. A cross-national cohort study reported that the prevalence of major depressive episode in Japan is lower than that in other countries.^30^ Consequently, it is plausible that racial disparities, including the social environment, may have contributed to the differences in the rates of depression.

Overall, the results of the present showed that FIM scores at discharge and FIM efficiency were significantly lower in patients with depression compared with those without. These findings are consistent with those of previous studies,^7^, ^8^, ^9^, ^10^ which showed an association between depression and poorer functional outcomes. Notably, however, FIM gain was not statistically different between groups in the present study. This finding was not consistent with the findings of a previous study,^10^ which showed that the FIM gain was lower in patients with depression. This result may be attributed to the prolonged length of hospitalization in the depression group. The previous study^10^ did not report a significant difference in length of hospital stay between patients with and without depression. Thus, our findings suggest that depression affected the efficiency of functional recovery; however, the gain in FIM was not different. Despite suboptimal efficiency, equivalent outcomes may be attained over an extended period. Regarding the low rate of discharging home in the depression group, we hypothesize that this might be primarily related to the fact that the FIM scores at discharge were significantly lower in the depression group than in the non-depression group. However, we cannot rule out the possibility that environmental factors, such as the status of family and socioeconomic issues, were involved in both the development of depression and difficulties of discharging home.

In the present study, only 1 patient (9.1%) in the depression group was prescribed antidepressants. Available evidence suggests that pharmacologic interventions and psychological therapy may prevent depression and improve mood after stroke, although the evidence is only very low‐certainty.^31^ In well-controlled efficacy trials, antidepressants improved functional outcomes^32^^,^^33^ and reduced mortality^34^; thus, the use of antidepressants is recommended in clinical guidelines.^35^^,^^36^ However, cohort studies, which may better reflect the real-world clinical settings, showed that the rate of antidepressant use was not as high, ranging from 6.3% to 35%^7^^,^^10^^,^^12^ among those indicated to have post-stroke depression based on screening tools. Furthermore, a previous systematic review^37^ pointed out that the patients with post-stroke depression were inadequately treated. Thus, there is some evidence to suggest a practice gap between the clinical settings and evidence for post-stroke depression treatment. Future studies are required to explore the factors that may impede the implementation of pharmacologic therapies in clinical practice. In addition, future investigations should further evaluate the effect of interventions, such as pharmacologic therapy, on functional outcomes using rigorous research methodologies.

### Study limitations

This study has some limitations. First, it was a single-center, retrospective study conducted in Japan, and thus, the generalizability of these results to other countries and institutions should be considered cautiously. Second, we were unable to obtain a detailed depression status at discharge. Therefore, the present study only revealed the association between depression at admission and functional outcomes at discharge. Third, the sample size was fairly small. Nevertheless, despite such limitations, there are few reports of the prevalence of post-stroke depression in a rehabilitation ward and the relation between functional improvement and outcomes. As such, even with the small number of patients, we believe that the present report provides valuable clinical insights, could contribute to future studies through further case accumulation, and could lead to important findings on the rehabilitation of patients with post-stroke depression. Furthermore, we could not perform multiple regression analyses to adjust for the effects of other potentially associated factors for functional outcomes. Fourth, we excluded patients who could not complete the MINI due to cognitive impairment or aphasia. Further prospective studies with larger samples are required to confirm the effect of depression on the outcomes and to elucidate long-term functional outcomes, quality of life, and the relation between admission and discharge status in depression.

## Conclusions

Overall, the results of the present study showed that patients with stroke with depression in the convalescent rehabilitation ward had poorer efficiency of functional recovery. Future studies are required to show the effectiveness of early interventions on the efficiency of functional recovery in patients with stroke manifesting depression in rehabilitation wards.

## Suppliers

a. G*power software (ver. 3.1.9.6; Heinrich-Heine-Universität Düsseldorf, Düsseldorf, Germany)
b. R version 4.1.0; R Foundation for Statistical Computing
